# Building Public Health Quantitative Methods Capacity and Networks in sub-Saharan Africa: An Evaluation of a Faculty Training Program

**DOI:** 10.9745/GHSP-D-22-00507

**Published:** 2025-08-14

**Authors:** Oleosi Ntshebe, Sarah Anoke, Jesca M. Batidzirai, Chris Guure, Beatrice Muganda, Marcello Pagano, Muhammed Semakula, Elysia Larson

**Affiliations:** a Department of Population Studies, University of Botswana, Gaborone, Botswana.; b Department of Biostatistics, Harvard T.H. Chan School of Public Health, Boston, MA, USA.; c School of Mathematics, Statistics and Computer Science, University of KwaZulu-Natal, Pietermaritzburg, South Africa.; d Department of Biostatistics, School of Public Health, University of Ghana, Accra, Ghana.; e Partnership for African Social and Governance Research, Nairobi, Kenya.; f Rwanda Biomedical Center, Research Innovation and Data Science Division, Kigali, Rwanda.; g I-BioStat, Hasselt University, Hasselt, Belgium.; h Centre of Excellence in Data Science, Bio-statistics, University of Rwanda, Kigali, Rwanda.; i Department of Obstetrics and Gynecology, Beth Israel Deaconess Medical Center, Boston, MA, USA.; j Department of Obstetrics, Gynecology, and Reproductive Biology, Harvard Medical School, Boston, MA, USA.

## Abstract

**Introduction:**

There is a shortage of individuals trained in using quantitative methods in biomedical research in sub-Saharan Africa (SSA). Improving public health in SSA requires new ways to promote quantitative knowledge and skills among faculty in biomedical research and better-integrated network systems of support.

**Methods:**

We describe the development, implementation, and evaluation of an innovative faculty training and support program in SSA from December 2017–June 2020, using courses in monitoring and evaluation, data management, and complex surveys as prototypical examples. Indicators were selected to follow the 4 levels outlined in the Kirkpatrick evaluation model: reaction, learning, behavior, and results. We used survey data from faculty fellows and students and reported median change and interquartile ranges (IQR).

**Results:**

The training program created an international community of 26 faculty members working collaboratively to lead the training of 3 quantitative methods courses. The program increased faculty members’ knowledge of the course content (median increase 17 percentage points [IQR: 0, 20]). Faculty members, in turn, trained 380 students at institutions of higher education in 8 SSA countries (Botswana, Ethiopia, Ghana, Nigeria, Rwanda, South Africa, Tanzania, and Uganda).

**Conclusion:**

The program relied on collaborative funding from participating institutions and focused on individual capacity-strengthening. In the future, the program will be scaled to include other emerging areas, such as data science, will integrate institutional support and feedback, and will move some of the training and mentoring activities to an online platform. Finally, to ensure that faculty have both improved confidence and improvement in competence, in future iterations, the program will include competency evaluation at the start and end and pair fellows who need additional training with those who excelled to co-teach.

## Abstract (precis)

Capacity-strengthening for faculty teaching quantitative skills can be accomplished through a cross-national training program that simultaneously builds research networks.

## Teaser

**Key Findings:**

- The faculty training program supported 26 fellows and strengthened collaborative links among the academic staff at 8 institutions of higher learning in sub-Saharan Africa.
- The fellows subsequently conducted trainings at their home institutions, which resulted in 380 students participating in a quantitative methods research course.

**Key Implications:**

- The faculty training program is an opportunity for cross-national train-the-trainer models to strengthen both capacity in quantitative methods and cross-national educational and research collaborations.
- As the current program evolved to center training in sub-Saharan Africa, it could continue to evolve to focus on regional opportunities that could reduce the dependence on outside funding and strengthen local partnerships.
- Training organizers should work with fellows’ home institutions in sub-Saharan Africa to reduce structural barriers to faculty growth and subsequent implementation of the training program.

## INTRODUCTION

There is a dearth of trained quantitative researchers in higher education in sub-Saharan Africa (SSA).^1^ While evidence suggests an increase in research outputs and health-related publications from authors in SSA, this comes from a small number of countries, and authors from high-income countries still dominate most public health publications that use data from SSA.^2^^–^^4^ This perpetuates power imbalances, as decisions made about defining research questions, what data to collect, and how to analyze these data are made by individuals removed from the communities addressed. Increased epidemiology and public health training have been associated with increased research output.^4^

Past efforts on capacity-strengthening in public health in SSA emphasized statistical training, developing institutional capacity to conduct health research, and improving fragile health systems.^4^^,^^5^ Other initiatives focused mainly on knowledge generation within universities and research networks, with little attention to the design of questions that resonate with national policy and individual talent development as an initiative’s primary objective.^6^ Recent initiatives, such as operational research in Rwanda, appeal to training and mentoring researchers to lead research projects from initial idea to publication of research findings.^7^ Moreover, countries such as South Africa now lead in research investments to improve local research capacity and strengthen international research collaboration and national research systems.^3^^,^^4^^,^^8^

Redesigning capacity-strengthening and training initiatives in public health in SSA is necessary and timely to promote globally competitive research and address the region’s public health challenges.^1^^,^^3^^,^^4^ These training types present prospects for future faculty training programs and collaborations with continuity and sustainability elements for strengthening biostatistical capacity in public health in low- and middle-income countries.

Redesigning capacity-strengthening and training initiatives in public health in SSA is necessary and timely to promote globally competitive research and address the region’s public health challenges.

We describe a faculty training program that aimed to support junior faculty in SSA to improve their pedagogical abilities, confidence in teaching, and content knowledge specific to quantitative methods courses and develop a cross-institutional and cross-country peer network that can lead to support future teaching and research collaborations. By providing didactic and experiential learning, the program was meant to improve faculty’s ability to teach students and implement these courses at their institutions, leading to improved capacity in quantitative methods for their students. In addition, the collaborations aimed to foster research proposed and conducted by the faculty, ultimately improving the quality and quantity of health research led by researchers in SSA. We describe the approach, outcomes, and provide lessons learned while implementing curricula on 3 courses with faculty from universities across 8 countries (Botswana, Ethiopia, Ghana, Nigeria, Rwanda, South Africa, Tanzania, and Uganda) in SSA.

## FACULTY TRAINING PROGRAM DESCRIPTION

From 2017–2020, the McGoldrick Professional Development Program in Public Health offered 3 in-person faculty training courses on quantitative methods for monitoring and evaluation (M&E), methods for data management with software application (DM), and complex survey analysis (CSA). In 2021, the program offered a virtual faculty training course on health data science (HDS). Faculty (including postdoctoral fellows, instructors, and lecturers) who had experience with the course content area and could teach the course at their home institution in sub-Saharan Africa were eligible to apply for the program as faculty fellows. The application required a letter of recommendation from a faculty mentor that needed to include a statement of institutional support to offer the course at their institution during the following academic year.

### Initial Training Model

M&E was the first course offered by the professional development program. The initial model consisted of a 1-week in-person workshop in Boston, USA, in December 2017, during which time the fellows met with senior faculty at Harvard T.H. Chan School of Public Health and were introduced to the M&E course syllabus, reviewed complex course content, and received pedagogical training. As part of the pedagogical training, faculty fellows were given opportunities to do a practice teaching session and receive feedback from their colleagues and senior faculty. After this workshop, the course director (who was based at Harvard T.H. Chan School of Public Health) traveled to each of the fellows’ home institutions to assist them in implementing the first round of the course for their students.

### Model Shifted to Center in sub-Saharan Africa

After implementation of the M&E course, discussions were held between programmatic staff and faculty fellows about changes that could be made to the model for future courses. For the DM and CSA courses, the model was adapted to be centered in SSA and increase opportunities for international collaboration. Training and implementation occurred in 4 phases over 10 months: an online training platform, an in-person workshop, an inaugural course co-taught by all faculty trainees at the host institution, and home institution implementation supported, when possible, by the faculty fellows from other countries either in-person or virtually.

Through the online training platform, faculty fellows were introduced to course content that included 5 modules for each course. Each module included a prerecorded lecture, a hands-on activity, and a knowledge check. Depending on their preexisting knowledge of the course content, this phase took approximately 10–40 hours to complete. Feedback was provided in real time from the course director. In addition, faculty fellows were introduced to each other and communicated and exchanged ideas and experiences through online discussion boards.

Subsequently, the faculty fellows convened for a week-long in-person workshop in Rwanda (DM in 2019) or Ethiopia (CSA in 2020). During this workshop, activities included practice teaching with peer feedback, codevelopment of course assignments, and learning the more complex aspects of the course content.

Then, the faculty fellows co-taught the course to students at the host institution in Rwanda for DM and Ethiopia for CSA in what we refer to as the “inaugural course.” This phase gave them experience teaching the content in a low-risk environment where they had support from the course director and other fellows. At the end of the week-long inaugural course, the faculty fellows participated in a facilitated group discussion to reflect on the strengths of the course implementation, challenges, and support they anticipated needing to successfully implement at their home institution.

Finally, faculty fellows returned to their home institutions to adapt the course to their local context and then offer it to students at their institution. During this phase, faculty fellows from other institutions traveled to support faculty fellows’ implementation of the course. For example, a fellow from Uganda traveled to Botswana to support the implementation of the DM course.

### Model Shifted to Accommodate COVID-19 Pandemic Travel Restrictions

In 2021, for the HDS course, institutional travel restrictions during the COVID-19 pandemic prevented implementation of the previous training models. Therefore, we implemented a modified approach with asynchronous training via the online platform, followed by a week-long virtual workshop, and finally, a week-long inaugural course that was held virtually.

### Evaluation Methods

Program evaluation indicators were selected to follow the 4 levels outlined in the Kirkpatrick evaluation model: reaction, learning, behavior, and results.^9^ For the HDS course, only reaction indicators and programmatic data were collected. We used survey data conducted with the fellows before and immediately following the training to measure their reaction and learning and data collected in May 2020 (4 months–2.5 years after completion of training) to determine behavior and results. To measure results from the students’ perspectives, we conducted a survey in June 2020 with students who took the M&E course in 2018. Data were collected using SurveyCTO and analyzed using Stata version 17 (StataCorp, College Station, TX, USA). Indices for knowledge, perceived knowledge, and confidence were scaled to range from 0 to 100. We reported descriptive statistics for each indicator of interest and then used the Wilcoxon matched-pairs signed-rank test to assess the changes in confidence and knowledge from before to after the in-person training. We used thematic analysis to analyze open-text responses.

### Ethical Approval

This evaluation received non-human subjects determination from the Harvard T.H. Chan School of Public Health.

## RESULTS

### Faculty Fellow Training

A total of 26 faculty fellows representing institutions of higher learning in 8 African countries (Botswana, Ethiopia, Ghana, Nigeria, Rwanda, South Africa, Tanzania, and Uganda) participated in the in-person faculty training program from 2017 to 2020, with 7 to 11 fellows trained each year. Fellows were trained to teach either 1 course (18 fellows), 2 courses (5 fellows), or 3 courses (3 fellows). Fellows included junior researchers (4; 15%), junior faculty (11; 42%), and senior faculty (11; 42%) who had demonstrated interest in public health research and education. Of the fellows, 18 (69%) were male and 8 (31%) were female.

The fellows’ reaction to the training was positive, with 96% saying they would definitely recommend the workshop training to another faculty fellow (Table 1). For fellows in either the DM or CSA course, the median increase in confidence in pedagogy was 29 percentage points (IQR: 20, 44); median increase in confidence in teaching the course content area was also 29 percentage points (IQR: 19, 42); and median increase in fellow’s knowledge was 17 percentage points (IQR: 0, 20). Fellows’ confidence in teaching the course content after the training was not associated with their knowledge level of the course content after training.

**TABLE 1.** Indicators of Training Reaction, Learning, Behavior, and Results Among the Faculty Fellows

	**Full Cohort** ^a^	**M&E (N=7)**	**Data Management (N=11)**	**Complex Survey Analysis (N=9)**	**Health Data Science (N=8)**
**Reaction**					
Rating of overall quality of course content, no. (%)					
Excellent	16 (57)		6 (55)	6 (67)	4 (50)
Very good	11 (39)		5 (45)	3 (33)	3 (38)
Average, fair, or poor^b^	1 (4)		0 (0)	0 (0)	1 (12)
Rating of overall usefulness of course content					
Excellent	16 (57)		8 (73)	4 (44)	4 (50)
Very good	12 (43)		3 (27)	5 (56)	4 (50)
Average, fair, or poor^b^	0 (0)		0 (0)	0 (0)	0 (0)
Would recommend workshop training to another faculty fellow, no. (%)					
Definitely would	27 (96)		11 (100)	8 (89)	8 (100)
Probably would	1 (4)		0 (0)	1 (11)	0 (0)
Probably would not or definitely would not^2^	0 (0)		0 (0)	0 (0)	0 (0)
**Learning, median (IQR)**					
Change in confidence in teaching course content^c^	29 (19, 42)		24 (19, 48)	33 (8, 33)	—
Change in confidence in teaching pedagogy^c^	33 (22, 47)		33 (0, 44)	25 (20, 50)	—
Change in knowledge for fellows in content area (quiz)^d^	20 (0, 20)		20 (0, 40)	0 (0, 20)	—
**Behavior**					
The McGoldrick program has impacted my work or teaching, no. (%)	21 (100)	7 (100)	11 (100)	9 (100)	
**Results**					
Has offered the course at their institution, no. (%)	14 (52)	7 (100)	6 (55)	1 (11)	—
Among those who have offered the course					
Times course was offered, median (IQR)	1 (1, 2)	1 (1, 2)	1 (1, 2)	1 (1,1)	—
Total students reached, no.	380	228	139	13	—
Students reached per offering, median (IQR)	21 (11, 25)	22 (21, 25)	14 (11, 21)	13 (13, 13)	—
Course format, no. (%)					
Short course	16 (84)	8 (89)	6 (67)	1 (100)	—
Full semester course	2 (11)	1 (11)	2 (22)	0 (0)	—
Incorporated content into existing course, no. (%)	1 (5)	0 (0)	1 (11)	0 (0)	—
Sessions, median (IQR)	14 (10, 19)	18 (10, 24)	10 (9, 15)	14 (14, 14)	—
Faculty, median (IQR)	4 (2, 6)	5 (4, 6)	2 (2, 3)	9 (9, 9)	—
Faculty from outside institutions, median (IQR)	1 (0, 1)	1 (1, 1)	0 (0, 1)	7 (7, 7)	—

Abbreviations: IQR, interquartile range; M&E, monitoring and evaluation.

^a^ This is the full cohort for which data are available, which could represent participants from 1, 2, 3, or 4 rounds of training.

^b^ Response categories combined for reporting purposes.

^c^ Summative index of questions with 4- or 5-level response options, scaled from 0–100 so that the difference represents a percentage point change.

^d^ Number of questions correct on 5-question quiz scaled from 0–100 so that the difference represents a percentage point change.

As a result of participating in the faculty training program, faculty fellows increased their confidence in pedagogy and teaching the course content, as well as their knowledge.

In open-ended responses after the training, many fellows commented on a desire to continue collaborations after the program had finished.

*I would like this cohort of trainees to remain in a network and collaborate on more projects.*

*I would suggest we keep helping each other out especially with regards to the data collection software.*

Several fellows either suggested that the length of the training program be extended or that support be continued after the workshop was completed. Many fellows commented on how much they had learned.

*I learned a lot. Even if the primary goal of the training is data management using software application, I learned so much about teaching skill.*

### Student Training

In subsequent offerings of the courses at fellows’ home institutions, fellows reached 380 students, incorporating support from either the course director or another faculty fellow in person 12 of 19 times (63%). They adapted the content to fit their context by adapting the number and length of sessions, sometimes incorporating the content into an already existing course or removing some content from the curriculum; providing additional instruction time for specific sessions to meet the needs of the student population; and including local examples. When asked what support fellows needed to implement the course at their university, nearly every fellow requested one of the other fellows to co-teach the course with them. COVID-19 pandemic changes to education affected the implementation of the courses after March 2020. The HDS course did not receive support from the McGoldrick program to have fellows travel to co-teach. The fellows did not implement the HDS course at their home institutions within the year following the training and workshop.

Faculty fellows from 5 of the 6 institutions offering the M&E course emailed an anonymous follow-up survey to students approximately 1 year after course completion. Of the 150 students contacted, 62 completed the survey (response rate: 40%). Students who took the M&E course found it to be useful and interesting (Table 2). They described many ways in which they incorporated what they learned into their work. Some common themes included conducting and interpreting quantitative analysis, using Stata software, formulating research questions and hypotheses, improving sampling for evaluation, and developing project indicators. Six students said they had not incorporated changes, citing either not having an opportunity to do so or not having sufficiently advanced or practical skills to do so. In the additional comments section of the survey, the most common suggestion on how to improve the course was to extend the length of the course. Other common themes included incorporating more practical examples with in-class time to practice/apply learning and using additional kinds of analytic software.

**TABLE 2.** Survey Results From Students Participating in the First Round of Monitoring and Evaluation Courses Offered at Faculty Fellows’ Home Institutions

	**No. (%)** **(N=62)**
Responded “yes” the training was worth their time	60 (97)
Words selected to describe the course content^a^	
Useful	47 (76)
Not useful	0 (0)
Challenging	17 (27)
Easy	0 (0)
Interesting	40 (65)
Innovative	19 (31)
Stale	0 (0)
Rating of the delivery of the course^b^	
Excellent	20 (32)
Very good	31 (50)
Average	10 (16)
Fair	1 (2)
Poor	0 (0)
Self-report of how much skills improved as a result of taking this course	
A large amount	26 (42)
A moderate amount	31 (50)
A small amount	5 (8)
Not at all	0 (0)
Introduced changes into work or research after course	53 (85)

^a^ Respondent could select as many as they wanted from the list provided.

^b^ Delivery was defined as the course instruction and implementation.

### Development of Institutional and Cross-Country Collaborations

The professional network built by the program has led to additional collaborations for grant applications between faculty fellows. In one, a collaboration between Harvard University and University of Ghana faculty members developed a Master of Science Biostatistics program at the University of Ghana. Others were a data science training program in SSA and a needs assessment to develop a training program in epidemiology and biostatistics in TB in SSA.

### Implementation Costs

Costs for each model of implementation varied widely. Costs were largely driven by flights and lodging for the in-person sessions. The cost of the program coordinator and faculty were provided in kind and not included in the following results. The initial model based in Boston was the most expensive, as each fellow flew from SSA to Boston, with an average cost per fellow of US$3,237. The SSA-centered model only required 2 trainers to fly between continents for the training while all fellows flew within SSA. While the in-person training was for 1 week in the initial model and 2 weeks for the SSA-centered model, total lodging prices were similar, given the different costs between countries. Despite the longer length of training, costs for the SSA-centered model were still lower than the initial model, at US$1,948 per fellow for the first year and US$2,858 per fellow the other year. For the final phase of the model where fellows taught the course at their home institutions, costs were again significantly lower for the SSA-centered approach as flights were within the continent instead of from Boston (US$2,409 versus US$3,377). The cost of the all-virtual model only included the time of the program coordinator and faculty teaching the course.

## DISCUSSION

This faculty training program intentionally evolved to center the program within SSA by providing in-person training at a host institution in SSA and teaching support from within the network of African faculty instead of faculty from the United States. These efforts helped shift intellectual and logistical decision-making to local institutions and further supported the growth of a peer network.

The program has successfully graduated 26 fellows who continue to work collaboratively. The program combines evidence-based training techniques, including online education, group discussions, didactic and experiential learning, and network support.^10^ The pedagogical skills, improved confidence in teaching, and collaboration/support network are likely to reach beyond the individual courses offered.

The program has successfully graduated 26 fellows who continue to work collaboratively.

### Challenges and Opportunities

There were several challenges and opportunities with the program. Because the program focuses on individual faculty, it had little impact on structural barriers to faculty growth and implementation at their home institutions. There were 2 opportunities to affect structural barriers within the current program. First, faculty who traveled to another site for the training were provided a reprieve from administrative tasks at their home institution and were better able to focus on the training. Second, in the application, we required a letter of institutional commitment to support the fellow in offering the course at the home institution. We saw this commitment during implementation in the form of protecting faculty time, providing physical space and computer facilities to offer the course, and providing administrative support for implementation.

Another identified challenge is that some faculty fellows may have had increased confidence in teaching specific course content even if they did not have the knowledge to effectively teach. If these faculty are not adequately supported, this could lead to poor training of students at their home institution.

In our evaluation, we had a low survey response rate from students. If students who gained more from the program were more likely to respond, this may have biased our results to look more favorable.

One additional opportunity for faculty education programs such as this one is to assist in closing gender inequities in higher education. The gender distribution of faculty fellows in this project represents that of the programs they were drawn from but missed an opportunity to recruit faculty through a stratified approach that would ensure gender equity in this training opportunity.^11^

A final opportunity identified through the evolution of the model to be centered in SSA was an opportunity to combine research and evaluation with training opportunities to reduce costs. The faculty teaching the CSA course were already traveling to Ethiopia for research and able to offer the course without the cost of an additional trip. Future programs could further capitalize on existing research and evaluation programs to host training opportunities among research faculty.

### Next Steps

The materials for the 4 courses are now available to researchers and educators. A critical aspect of the implementation model for all courses was teaching support for faculty fellows when they returned to their host institution. In future iterations, the program is exploring ways to work with home institutions to protect faculty fellows’ time from administrative tasks during this time. Learning from this program and the success of the Demographic and Health Surveys Faculty Fellows Program,^12^ our program will support faculty fellows trained from the same institution together with a common senior leader at the institution to work together to implement their courses.

The program will continue to move forward with an SSA-centered model with further focus on region-specific training and support. In addition, plans for the program include moving some of the training and mentoring activities online to reduce costs and benefit more users in SSA. However, while online learning significantly reduced the costs, we found that it also led to lower implementation of the training at fellows’ home institutions. Fellows in the online course had fewer opportunities to network with each other, resulting in fewer collaborations on research and grants external to the training program. We believe that moving the program entirely online is unlikely to be successful.

To ensure that faculty leave the training with both improved confidence and linked improvement in competence, future program iterations will include additional pre-training for fellows entering without sufficient content knowledge and additional training and support for fellows completing the course without sufficient content knowledge. Finally, fellows will be intentionally paired with others to balance individual strengths and weaknesses to co-teach when the course is offered at their home institution.

While the current approach for individual capacity-strengthening for quantitative methods often focuses on brief, 3–5 day long workshops that focus on the course content, this evaluation has demonstrated that longer, more involved capacity-strengthening programs are likely to be effective. They offer the additional benefit of building a network of educators and researchers and giving time and opportunity for pedagogical training.

## CONCLUSION

This faculty fellow training program has demonstrated that it is possible to create an international capacity-strengthening program to support faculty and develop leaders for quantitative methods expertise at institutions of higher learning in SSA. With suitable investments, this faculty training program can provide an adequate supply of highly skilled academics in SSA to deliver training in quantitative topics. Finally, home institution engagement strategies will be important to ensure individual fellows’ learning and future practice to implement the teaching of quantitative research at institutions of higher education in SSA.

## Notes

**Peer Reviewed**

**First Published Online:** January 16, 2025.
